# VDR, SOD-2, and CYP24A1 Gene Expression in Different Genotypes of BsmI SNP of the Vitamin D Receptor Gene in Individuals with Hypovitaminosis

**DOI:** 10.3390/nu15163565

**Published:** 2023-08-13

**Authors:** Vanessa Rosa Retamoso, Fernanda Barbisan, Graziele Meira Moro, Patricia Maurer, Débora Vasquez Rubio, Lauren Flores Viera dos Santos, Lyana Berro Feijóo, Matias Nunes Frizzo, Ivana Beatrice Mânica da Cruz, Vanusa Manfredini, Ana Letícia Vargas Barcelos, Jacqueline da Costa Escobar Piccoli

**Affiliations:** 1 Postgraduate Program in Biochemistry, Federal University of Pampa e Campus Uruguaiana, BR 472-Km 592-Mailbox 118, Uruuguaiana 97508-000, RS, Brazil; patytm@gmail.com (P.M.); debora.v.rubio@gmail.com (D.V.R.); lyanaberro78@hotmail.com (L.B.F.); vanusa_manfredini@yahoo.com.br (V.M.); analeticia@unipampa.edu.br (A.L.V.B.); 2 Pharmacy Department and Post Graduation in Gerontology, Federal University of Santa Maria (UFSM), Santa Maria 97105-900, RS, Brazil; fernandabarbisan@gmail.com (F.B.); graziela.moro@acad.ufsm.br (G.M.M.); ibmcruz@hotmail.com (I.B.M.d.C.); 3 Physiotherapy Course, Federal University of Pampa e Campus Uruguaiana, BR 472-Km 592-Mailbox 118, Uruguaiana 97508-000, RS, Brazil; laurenfsantos16@gmail.com; 4 Department of Life Sciences, Northwest Regional University (Unijui), R. do Comércio, 3000-Universitário, Ijuí 98700-000, RS, Brazil; matias.frizzo@gmail.com; 5 Nutrition Course, Federal University of Pampa, Campus Itaqui, Road Luiz Joaquim de Sá Brito, Itaqui 97650-000, RS, Brazil

**Keywords:** vitamin D, hypovitaminosis, cytochrome P450 (CYP), SOD2

## Abstract

Background: Hypovitaminosis D is a public health problem due to its implications for various diseases. Vitamin D has numerous functions, such as modulating the metabolism of cellular tissues, and it is expressed through the vitamin D receptor (VDR) gene that may influence gene expression modulation, which plays an important role in vitamin D metabolism. Objective: To evaluate the effect of the genotypes of BsmI single nucleotide polymorphism (SNP) of the VDR gene on VDR, SOD2, and CYP24A1 gene expression in individuals with low serum vitamin D levels. Methods: This was a cross-sectional analytical study. After signing the informed consent form, individuals were invited to participate and answered a structured questionnaire with identification data. Blood was collected for biochemical analysis, and vitamin D was measured by chemiluminescence; BsmI polymorphism was determined using real-time polymerase chain reaction (PCR) assays with TaqMan allelic discrimination, and gene expression was conducted by qRT-PCR using QuantiFast SYBR^®^ Green PCR Master Mix. Data were analyzed using the SPSS 20.0 software, and differences were considered significant at *p* < 0.05. Results: 98 individuals with vitamin D ≤ 20 ng/dL were evaluated, and the BsmI SNP of the VDR gene showed CYP24A1 overexpression and low SOD2 expression. Conclusion: BsmI SNP of the VDR gene can modulate the expression of the genes evaluated without interfering with serum levels.

## 1. Introduction

Vitamin D is a steroid hormone of the fat-soluble vitamin class involved in various biological processes, including cell proliferation, bone metabolism, and cell differentiation [1]; it is also responsible for a complex multi-step metabolism and acts as a hormone in numerous extra-skeletal targets [2]. Vitamin D deficiency, which occurs when vitamin D levels in the blood are below 20 ng/mL, is prevalent around the world [3], mainly affecting countries with little sun exposure due to climatic conditions, high latitudes, and winter regimes, in addition to other conditions (e.g., skin hyperpigmentation and chronic diseases) and affecting eating habits, pregnancy, breastfeeding [4]. This condition is considered a global health problem, as low vitamin D levels are associated with an increased risk of various diseases and metabolic changes [5]. In Brazil, the Brazilian Society of Endocrinology and Metabology recommends serum levels above 20 ng/mL for the general healthy population and 30–60 ng/mL for at-risk groups such as the elderly, pregnant women, patients with osteomalacia, rickets, osteoporosis, secondary hyperparathyroidism, pre-bariatric patients, and inflammatory, autoimmune, and chronic kidney diseases [6]. 

The circulating effects of 1,25(OH)_2_D are mediated by the vitamin D receptor (VDR), which is a member of the superfamily of intracellular nuclear receptors [7]. Vitamin D also has genomic effects, including cell apoptosis regulation, differentiation, proliferation, DNA repair, oxidative stress, and cell metabolism, which are driven by transcription factors [1].

Hence, VDR and vitamin D metabolic enzymes are expressed in all innate and adaptive arms of the immune system, and genomic approaches for gene expression profiling have identified various VDR-regulated genes implicated in regulating innate and adaptive immunity, including CYP and manganese-dependent superoxide dismutase (SOD2) genes [8]. Genetic variations called single nucleotide polymorphisms (SNP) can reach the human genome and alter different genes’ transcription and translation steps. The VDR gene is located on chromosome 12q13.1. It has over 900 allelic variants in the VDR locus, and the most studied polymorphisms of the VDR gene are Apal (rs7975232), BsmI (rs1544410), Taql (rs731236), and Fokl (rs10735810); the ApaI, TaqI, and BsmI SNPs are silent genetic variants that increase mRNA stability [9]. This study will address BsmI (rs 1544410), which is located in intron 8, and the result of substituting an adenine-guanine (A-G) [10]. Researchers have associated BsmI with osteoarthritis [11], breast cancer [12], melanoma [13], and system lupus erythematosus [14], among others, although data on its association with serum vitamin D levels are still conflicting [14,15].

Other genes are also known to modulate the expression of the VDR gene. For instance, one study found that the CYP gene, whose expression was constant, is regulated by fasting in the liver. Notably, adipose tissue and the brain are the organs where vitamin D seems to play an important, albeit not fully known, role [3]. In addition, the SOD2 gene has an antioxidant function, which may be relevant at low serum vitamin D levels [16]. Therefore, it is important to shed more light on how the different genotypes of the BsmI SNP of the VDR gene can modulate the expression of the genes of the VDR itself and others in its pathway. 

Given the above, this study sought to evaluate the effect of the genotypes of BsmI SNP of the VDR gene on the expression of different genes (VDR, SOD2, and CYP24A1) in individuals with low serum vitamin D levels.

## 2. Materials and Methods

### 2.1. Experimental Design 

This was an epidemiological, analytical, observational cross-sectional study in which inflammatory, biochemical, and genetic marker levels were assessed in individuals with hypovitaminosis D. 

### 2.2. Study Population and Design

**Figure 1:**
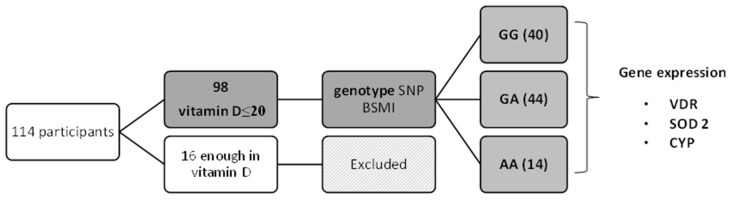
Participant flowchart.

The study was conducted with adults (18–59 years of age) in Rio Grande do Sul State (southern Brazil) from August to November (i.e., winter and spring). The volunteers signed an informed consent form and answered a questionnaire with their socioeconomic data and lifestyle. After fasting for 12 h, venous blood was collected for analysis. After obtaining the serum vitamin D values results, volunteers with measurements of up to 20 ng/mL continued in the study [17], and the others were excluded (Figure 1). Genotyping for BsmI SNP was performed, and afterwards, participants were divided into three groups according to genotypes: group GG (*n* = 40), group GA (*n* = 44), and group AA (*n* = 14). 

### 2.3. Genetic-Molecular Analysis

#### 2.3.1. DNA Extraction

Genomic DNA was isolated from peripheral blood leukocytes using the GFX Genomic Blood DNA Purification Extraction Kit (Amersham Biosciences Inc., Co., Amersham, UK).

#### 2.3.2. Gene Amplification

The BsmI polymorphism was determined using real-time PCR assays with TaqMan allelic discrimination (Applied Biosystems, Foster City, CA, USA) according to the protocol described by Retamoso et al. (2023).

### 2.4. Gene Expression by qRT-PCR

Modulation of gene expression was conducted by qRT-PCR analysis using an approach similar to that described in the literature [17]. Briefly, the total RNA obtained from each treatment was isolated using the TRIzol^®^ reagent and quantified using a NanoDrop™ 1000 Spectrophotometer System^®^ (Thermo Fisher Scientific, Wilmington, DE, USA). Then, a cDNA was obtained using the Script™ cDNA Synthesis Kit (Bio-Rad Laboratories, Hercules, CA, USA) according to the manufacturer’s instructions. qRT-PCR was performed on a Rotor-Gene Q 5plex HRM system (QIAGEN biotechnology, Hilden, Germany). A melting curve was generated from 60 °C to 90 °C in 0.5 °C increments for 5 s at each temperature. All reactions were performed in triplicate, with 1 µM of each primer and 2× QuantiFast SYBR^®^ Green PCR Master Mix; the final reaction volume was 20 µL. Specific forward and reverse primer sequences are listed in Table 1.

The β-actin housekeeping gene was used as an internal control of gene expression analysis. Relative gene expression was calculated using the comparative Ct method and expressed as fold expression relative to the control. 

#### Anthropometric and Physiological Assays 

The anthropometric measurements evaluated were weight, height, and waist and hip circumferences; the nutritional status was classified according to the BMI [18]. 

### 2.5. Biochemical Assays

Peripheral blood samples were collected after 12 h of fasting. The samples were centrifuged for 15 min at 3000 rpm, and aliquots of serum and plasma were stored at 20 °C for further analysis. The lipid profile, blood glucose, total cholesterol, high-density lipoprotein (HDL), triglycerides, gamma-glutamyl transferase, and transaminases were determined with a commercial kit (LABTEST) using automated equipment (ChemWell Labtest) and according to the manufacturer’s instructions. All quality control criteria were followed. Low-density lipoprotein (LDL) cholesterol levels were measured using the equation of Friedewald.

### 2.6. Vitamin D Analysis 

Serum vitamin D 25OHD_3_ was analyzed by high-performance chromatography (HPLC). The deficiency, insufficiency, and adequacy of 25OHD_3_ were classified according to the cut-off points of Maeda, 2014.

### 2.7. Statistical Analysis

Statistical analyses were performed using the SPSS statistical software (version 20.0). Quantitative variables were analyzed using the Student t-test or bivariate analysis of variance, followed by Bonferroni’s post hoc test. Categorical variables were analyzed using the chi-square test. The Hardy–Weinberg equilibrium was tested using the Chi-square test. The significance level considered was *p* < 0.05.

### 2.8. Ethics

This study complied with the ethical principles for research involving humans according to the Declaration of Helsinki and was approved by the Ethics Committee of UNIPAMPA (protocol number 977827). The participants’ privacy rights were respected, and all individuals signed an informed consent form to participate.

## 3. Results

Ninety-eight individuals with hypovitaminosis D (≤20 ng/dL), with a mean age of 30.5 ± 11 years, 54.1% female, and (54.1%) self-declared white, were recruited. Table 2 lists the baseline characteristics of the total sample.

Table 3 lists the descriptive analyses of anthropometric and biochemical measurements. 

The genotypic and allelic frequencies of the BsmI polymorphism of the VDR gene in the total sample are described in Table 4. The studied sample presented Hardy–Weinberg equilibrium (χ^2^ = 2.01). 

Comparisons between genotype groups are listed in Table 5, and no differences were found between the markers analyzed among the different BsmI genotypes.

**Figure 2:**
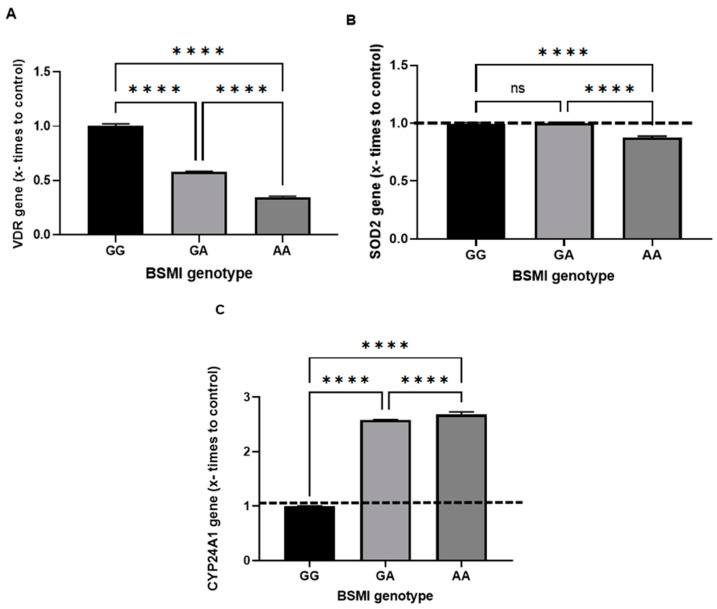
Comparison of the effects of BsmI on VDR, SOD2, and CYP24A1 gene expression. VDR (**A**), where *p* **** (0.001) among all BsmI genotypes: GGxGA, GGxAA, GAxAA. SOD2 (**B**) *p* **** (0.001) was among the BsmI genotypes: GGxAA GAxGG. CYP24A1 (**C**) *p* **** (0.001) among all BsmI genotypes: GGxGA, GGxAA, GAxAA SOD2 (**B**) and CYP24A1 (**C**) gene expression among PBMCs carrying different BsmI genotypes of the VDR gene. Data are presented as mean ± SD. Analysis was performed and compared by one-way analysis of variance followed by the Bonferroni post hoc test. VDR gene = vitamin D receptor; SOD-2 gene = superoxide dismutase 2; CYP24A1 gene = cytochrome P450 family 24 subfamily A member 1.

The VDR, SOD2, and CYP24A1 expressions were performed in each genotype group for the VDR BsmI SNP; the results are illustrated in Figure 2. VDR gene expression was significantly lower in the GA and AA genotypes than in the wild-type GG genotype, and AA showed the lowest expression (Figure 2A). The SOD2 gene was also significantly less expressed in the AA group than GG and GA, which showed no differences (Figure 2B). In contrast, CYP24A1 overexpression was observed in the GA and AA genotypes (Figure 2C).

## 4. Discussion

For the first time, this study demonstrated the expression of different genes in distinct genotypes of the BsmI SNP of the VDR gene (in addition to the VDR itself) in patients with hypovitaminosis D. The BsmI SNP negatively regulated the VDR and SOD2 gene and upregulated CYP24A1, thus showing the modulatory influence of BsmI on other genes in hypovitaminosis D.

The sample represents general characteristics observed in young and active individuals, most of whom were women, as observed in other studies (Table 2). The higher number of self-declared white individuals is due to a higher prevalence of self-declared white individuals in southern Brazil (i.e., the study region) [19]. As for the level of education, most individuals had incomplete higher education, implying that they could understand the questionnaire questions. This differs from the data found in the rest of Brazil, where a high proportion of people aged 25 years or older have only completed compulsory basic education (i.e., high school) [20]. In another study with individuals with vitamin D insufficiency and assisted by primary health care services, researchers reported serum vitamin D levels not being associated with the level of education, age, marital status, and income [21].

Another important finding is regarding the participants’ being overweight (Table 3), as the average BMI was within 27.1 kg/m^2^. A recent study evaluated serum vitamin D levels in healthy adult women and also showed that the nutritional status of the group evaluated was classified as overweight/obese, although without presenting any significant association [22]. Moreover, hormonal factors for the female participants (e.g., estrogen levels) may have also influenced VDR gene expression and even serum vitamin D levels. Despite not being the focus of this study, women with lower serum levels of 25-hydroxyvitamin D and higher tissue levels of VDR and tissue expression of the estrogen receptor gene had a significantly higher risk of breast cancer incidence [23].

In this study, the distribution of genotypes and alleles of BsmI SNP were in Hardy–Weinberg equilibrium, and there were no significant associations between BsmI genotypes and serum vitamin D levels or the other markers (Table 5), as previously demonstrated by Retamoso and collaborators [16]. In other reports, no significant differences between vitamin D levels or genotypic and allelic frequencies of polymorphisms in the VDR gene were found, even though cross-sectional studies have shown that VDR gene polymorphisms can reduce the affinity of the VDR for serum vitamin D levels [24]. Despite VDR genetic polymorphisms being determinants of vitamin D levels, they have other genetic and environmental factors that are influenced by sun exposure, diet, and even skin pigmentation [25].

Although food consumption was analyzed in a recent study [23], it is worth mentioning it because it is an environmental factor, given that the diet of Western countries has low vitamin D concentrations, which is the case in Brazil. In contrast, European countries consume the highest amounts of vitamin D, given that the population generally consumes fish and cod liver oil. In addition, there are also higher rates of vitamin D2 and vitamin D3 supplementation. Despite less sun exposure, other factors may increase vitamin D absorption compared to South American countries [26], even though it is known that the role of supplementation depends on the individual. The expression of the VDR gene is also influenced, as it is hypothesized that the response to vitamin D supplementation may be modulated by genetic variants in the VDR gene [9].

In order to clarify whether the BsmI SNP influences the expression of other genes related to the physiological role of vitamin D, VDR, CYP24A1, and SOD-2 gene expression was studied in the groups of the three genotypes. Thus, we know that the VDR can modulate the expression of various genes, and its inactivation occurs due to the lack or excess of vitamin D since the almost ubiquitous expression of the VDR gene corroborates data from the last 30 years showing that vitamin D not only regulates calcium homeostasis but also promotes immunity, growth, and cell differentiation [27].

The VDR gene was under-expressed in the GA and AA genotypes compared to GG and AA less than the others (Figure 2A). In another study that compared the frequency of GG versus AA and AG genotypes, the association with insufficient 25(OH)D concentrations was maintained, suggesting that BsmI, which regulates VDR expression, can modulate vitamin D levels in patients with cognitive disorders [23]. It is important to emphasize that this study did not evaluate specific pathological conditions but individuals with hypovitaminosis D, which may explain these findings. They were from different groups (people with hypovitaminosis D against a population with cognitive impairment) and there was a degree of genetic mixture in the population studied, considering that they are of different ethnic origins and have a high degree of miscegenation among the populations investigated.

Moreover, the SOD-2 gene was significantly less expressed in AA genotype carriers compared to GG and GA (Figure 2B). This result can be explained by the findings of Dauletbaev and collaborators [28], who investigated the impact of the genome on transcription by 1,25(OH)_2_D in carcinogenic cells. The 1,25(OH)_2_D induced genes such as SOD2, IRS2, BIRC3, and DUSP1/5 are cytoplasmic or mitochondrial signaling molecules that mediate the effects of growth factors and/or cytokine interactions with known anticancer properties. In other words, 1,25(OH)_2_D significantly induced mitochondrial expression but not cytosolic SOD2, which converts the free radical O2^•−^ (superoxide) into H_2_O_2_ to defend against free radicals [8]. 

Lastly, we evaluated whether the BsmI SNP of the VDR gene would modulate the expression of the CYP24A1 gene, which showed overexpression in carriers of the GA and AA genotypes compared to GG (Figure 2C), with AA being significantly more expressed than GG and GA, in which the mutated allele “A” possibly increases CYP24A1 expression. This is an unprecedented analysis in the literature, and the expression of most vitamin D target genes is 5× up- or down-regulated, meaning only a few genes respond with significant changes in vitamin D expression [29], as is the case of CYP24A1, which showed higher expression against BsmI SNP of the VDR gene. This may also influence one of the biological functions of the VDR (i.e., mRNA stability) and epigenetic changes (e.g., DNA methylation), which appear to modulate VDR gene expression [30]. 

Furthermore, it is important to consider that the physiological status of vitamin D is defined when its circulating levels are 25–70 ng/mL, in which the enzyme with 24-hydroxylase activity, CYP24A1, catalyzes the degradation reactions of 25(OH)D and 1,25(OH)_2_D. During this process, multiple hydroxylation reactions occur on the side chain, and CYP24A1 catabolizes 25(OH)D to prevent its eventual activation into 1,25(OH)_2_D and/or 1,25(OH)_2_D, for which CYP24A1 has higher affinity compared to 25(OH)D, to disrupt its biological activity [26]. Therefore, further research should be conducted to elucidate the role of BsmI SNP in different haplotype conditions with other polymorphisms of the VDR gene and determine the likelihood of it being in linkage disequilibrium with other alleles [31]. 

Despite our promising findings, it is important to mention some limiting factors of this study, including the participants’ levels of sun exposure, ethnic origin, skin color, and the use of biological markers (e.g., calcium, parathyroid hormone, and steroid hormones). Nevertheless, this study shed light on the genetic modulation of a VDR gene SNP in the hypovitaminosis D phenotype in humans, and future studies will better elucidate the genetic, epigenetic, and molecular points involved. Thus, despite the limitations, different genotypes of BsmI SNP of the VDR gene modulated the expression of the VDR, CYP24A1, and SOD2 genes even though they did not alter the circulating levels of vitamin D in individuals with hypovitaminosis D.

## Figures and Tables

**Table 1:** List of primers used and gene information.

Gene and Gene ID	NCBI Reference Sequence	Location	Size (pb)	Primers
VDR-7421	NG_008731.1	12q13.11	4.738	F-5′CCTTCACCATGGACGACATG3′ R-5′CGGCTTTGGTCACGTCACT3′
CYP24A1-1591	NG_008334	20q12	37449	F-5′CTCATGCTAAATACCCAGGTG-3′ R-5′TCGCTGGCAAAACGCGATGGG3′
SOD-2-6648	NG_008729	6q11	100,213	F-5′GCCCTGGAACCTCACATCAA-3′ R-5′GGTACTTCTCCTCGGTGACGTT3′

**Table 2:** Sociodemographic and lifestyle characteristics of the sample.

Characteristic	N (98)	(%)
Sex		
Female	53	54.1
Male	45	45.9
Self-declaration of color		
Black	20	20.4
Brown	25	25.5
White	53	54.1
Education		
Primary education (i)	5	5.1
Primary education (c)	1	1.0
Secondary education (i)	1	1.0
Secondary education (c)	16	16.3
Higher education (i)	53	54.1
Higher education (c)	20	20.4
Other (graduate degree)	2	2.0
Marital status		
Single	66	67.3
Married	25	25.5
Common-law marriage	6	6.1
Widowed	1	1.0
Performs physical activity		
Yes	48	49
No	50	51
Smokes		
Yes	10	10.2
No	87	88.8
Ex-smoker	1	1.0
Drinks alcohol		
Yes	63	63.3
No	35	35.7

c = complete, i = incomplete.

**Table 3:** Biochemical and anthropometric analysis of the sample.

Marker	Mean	±SD
Weight (kg)	77.8	19.0
Height (m)	1.8	0.16
BMI (kg/m^2^)	27.1	5.4
CC (cm)	88.2	14.7
QC (cm)	103.6	9.8
Total cholesterol (mg/dL)	177	47
HDL-C (mg/dL)	55.2	22.2
LDL-C (mg/dL)	94.9	41.2
Triglycerides (mg/dL)	146.1	142.2
Vitamin D (ng/mL)	17.03	4.04

BMI = body mass index, HDL-C = high-density lipoprotein cholesterol, LDL-C = low-density lipoprotein cholesterol, WC = waist circumference, HC = hip circumference.

**Table 4:** Allelic and genotypic frequency of BsmI SNP of the VDR gene.

BsmI SNP	Participants (*n* = 98)	(%)	* *p*-Value
Genotypic frequency			
GG	40	40.8	0.4
GA	44	44.9	0.46
AA	14	14.3	0.13
Allelic frequency			
Allele G	102	67.1	
Allele A	50	32.9	
Model			
AA + GA	54	55.1	
GG	40	44.9	

* Chi-square test.

**Table 5:** Comparison between the BsmI SNP genotype groups of the VDR gene and the variables studied.

	Genotype Group BsmI VDR			
	GG (40)	GA (44)	AA (14)	*p*
Age	29.8 ± 10.2	32.3 ± 11.4	29.8 ± 11.3	0.80
BMI (kg/m^2^)	26.8 ± 5.2	26.8 ± 5.3	28.6 ± 6.2	0.51
CC (cm)	87.46 ± 15.3	87.5 ± 12.7	92.6 ± 18.7	0.49
QC (cm)	103.36 ± 9	102.7 ± 9.6	107.36 ± 12.2	0.29
%fat	12.26 ± 10.9	12.6 ± 9.96	13.7 ± 11.0	0.90
Glucose (mg/dL)	91.24 ± 21.9	84.07 ± 22.1	92.6 ± 24.12	0.25
Total cholesterol (mg/dL)	182.5 ± 51.6	174.4 ± 47.3	169.8 ± 47.2	0.63
HDL-C (mg/dL)	58.16 ± 22.7	55.1 ± 22.6	45.34 ± 17.7	0.17
LDL-C (mg/dL)	95 ± 40.8	94.4 ± 44.8	96.17 ± 41.6	0.99
Triglycerides (mg/dL)	151.03 ± 149	135.91 ± 148.4	164.3 ± 104.8	0.78
Vitamin D (ng/mL)	17.45 ± 2.2	16.88 ± 4.3	16.34 ± 2.4	0.64

BMI = body mass index, HDL-C = high-density lipoprotein cholesterol, LDL-C = low-density lipoprotein cholesterol, WC = waist circumference, HC = hip circumference.
